# Navigating precision: the crucial role of next-generation sequencing recurrence risk assessment in tailoring adjuvant therapy for hormone receptor-positive, human epidermal growth factor Receptor2-negative early breast cancer

**DOI:** 10.1080/15384047.2024.2405060

**Published:** 2024-09-20

**Authors:** Ying Xu, Yingxue Qi, Zhongyu Lu, Yuan Tan, Dongsheng Chen, Haijun Luo

**Affiliations:** a Department of Obestetrics and Gynecology, Xiangyang Central Hospital, Affiliated Hospital of Hubei University of Arts and Science, Xiangyang, China; b The Medical Department, Jiangsu Simcere Diagnostics Co. Ltd. Nanjing Simcere Medical Laboratory Science Co. Ltd., The State Key Laboratory of Neurology and Oncology Drug Development, Nanjing, China; c Cancer Center, The First Affiliated Hospital of Jinzhou Medical University, Jinzhou, China; d Center of Translational Medicine, The First Affiliated Hospital of Jinzhou Medical University, Jinzhou, China; e Department of Pathology, The Affiliated Changsha Central Hospital, Hengyang Medical School, University of South China, Changsha, China

**Keywords:** Early breast cancer, HR-positive, HER2-negative, adjuvant therapy, recurrence risk assessment

## Abstract

Hormone receptor-positive (HR+)/human epidermal growth factor receptor 2-negative (HER2−) breast cancer is the most common subtype, representing over two-thirds of new diagnoses. Adjuvant therapy, which encompasses various medications and treatment durations, is the standard approach for managing early stage HR+ HER2- breast cancer. Optimizing treatment is essential to minimize unnecessary side effects while addressing the biological variability inherent in HR+/HER2− breast cancers. Incorporating biological biomarkers into treatment decisions, alongside traditional clinical factors, is vital. Gene expression assays can identify patients unlikely to benefit from adjuvant chemotherapy, thereby refining treatment strategies and improving risk assessment. This paper reviews evidence for several genomic tests, including Oncotype DX, MammaPrint, Breast Cancer Index, RucurIndex, and EndoPredict, which assist in tailoring adjuvant therapy. Additionally, we explore the role of liquid biopsies in personalizing treatment, emphasizing the importance of considering late relapse risks and potential benefits of extended systemic therapy for HR+/HER2− breast cancer patients.

## Introduction

Breast cancer (BC) is one of the most common cancers in women worldwide. It is highly heterogeneous and the various BC types differ from each other both morphologically and molecularly. Several known and unknown factors influence treatment response and the risk of relapse.^1–3^ Hormone receptor-positive (HR+) BC is a hormone-dependent tumor. Of the 300,000 patients diagnosed with breast cancer in 2023,^4^ approximately 190,000 were identified with early-stage HR-positive disease. HR+ BC progresses more slowly and has a better overall prognosis and longer survival period than certain other BC subtypes. The advantages of adjuvant chemotherapy in individuals diagnosed with HR+ BC early-stage breast cancer should be carefully weighed against its potential short- and long-term negative consequences. It is crucial to identify the specific patient population who are most likely to derive significant clinical benefits from chemotherapy. Endocrine therapy (ET) is one of the first targeted anticancer therapies to be clinically administered.^5,6^ Endocrine agents are the mainstay of early-stage ER+/HER2- BC treatment. Several large clinical trials demonstrated that 5 years of adjuvant hormonal therapy significantly reduces the risks of local recurrence, contralateral BC, distant recurrence, and death in patients with HR+ early-stage BC.^7,8^ Clinical trial data for patients with early-stage HR+/HER2- BC showed that combining adjuvant chemotherapy with ET significantly improves patient disease-free survival (DFS) and overall survival (OS) compared to Et al.one.^9,10^ Global initiatives are being made to ensure that women diagnosed with curable, early-stage breast cancer are provided with access to transformative technologies and treatments, such as genetic testing, essential biomarker analyses, and crucial therapeutics.The advancement of genomic assays has enabled the prediction of which patients with early hormone receptor-positive disease may derive benefits from chemotherapy in conjunction with endocrine therapy, thus avoiding unnecessary chemotherapy for many patients.

A major challenge inherent in the management of HR+ and especially HR+/HER2- early-stage BC is the risk of recurrence. After ≥5 years, it may be as high as ~ 50%. Clinical stage, tumor size, histological grade, and lymph node status all affect treatment efficacy and prognosis. Certain molecular states including the Oncotype DX**®** 21-gene recurrence score are now included in the eighth edition of the Joint Committee on Cancer (AJCC) staging guidelines.^11^ Biomarkers can identify the presence and/or progression of cancers, predict their responses to treatment, and play a key role in their clinical management.^12,13^ Multigene molecular testing tools capture certain molecular signatures and integrate them into risk scores.^14–17^ Other molecular detection technologies such as circulating tumor cells (CTCs) and ctDNA-minimal residual disease (MRD) liquid biopsy have demonstrated value in BC treatment and risk prediction.^18–21^

The present literature review assesses the value of molecular testing in adjuvant treatment and predicting the prognosis of HR+/HER2- early-stage BC, available evidence supporting the application of these techniques in various patient groups, and the extent to which these methods facilitate informed therapeutic decisions. It also evaluates the clinical utility of emerging biomarkers in the management of early adjuvant therapy against HR+/HER2- BC, addresses the hindrances to their implementation, and proposes pertinent future research directions.

## Application of various multigene detection methods in adjuvant chemotherapy for patients with stage T1–2N0–1 early invasive BC

### Oncotype DX®

The Oncotype DX Breast Recurrence Score**®** test was one of the first genomic assays approved for early-stage BC.^14^ The Oncotype DX**®** uses expression data from 21 genes (16 core genes and 5 reference genes, supplementary Table 1) to calculate a recurrence score (RS), which can be divided into three risk groups: high, intermediate, and low. It can be used to guide adjuvant chemotherapy or radiotherapy, especially in United States, Europe, Middle East, Africa, Japan and other regions(Table 1).

**Table 1.** Multigene assays for early-stage BC.

Multigene assays	Gene Number	Detection Method	Applicable Population	Risk stratification	Applications	Region	Samples	Guidlines
Oncotype DX®	21	RT-PCR	HR positive, HER2 negative BC，Stage N0–N1	Low risk RS < 11 Intermediate risk RS 11–25 High risk RS ≥ 26	Predict prognosis, Direct adjuvant chemotherapy or radiotherapy	United States, Canada; Europe, Middle East, Africa; Japan, Latin America	FFPE	NCCN/ASCO/ESMO/CSCO
MammaPrint®	70	DNA-Microarray	HR positive, HER2 negative BC，Stage N0–N1	Low genomic risk score −1 ~ 0 High genomic risk score 0 ~ 1	Predict the risk of recurrence and metastasis	United States, Canada，Spain, Middle East, etc	FFPE; Fresh frozen tissues	NCCN/ASCO/ESMO/CSCO
EndoPredict®	12	RT-PCR	HR positive, HER2 negative BC，Stage N0–N1	Low risk EP < 5 EPclin score < 3.3 High risk EP ≥ 5 EPclin score ≥ 3.3	Predict prognosis	Europe	FFPE	NCCN/ASCO/ESMO/CSCO
Breast Cancer Index®	11	RT-PCR	HR positive, HER2 negative BC，Stage N0–N1	HOXB13/IL17BR ratio and molecular grade index Low risk – BCI < 5.0825 Intermediate risk 5.0825 < BCI < 6.5025 High risk – BCI ≥ 6.5025	Predict prognosis, Predict response to endocrine therapy	United States	FFPE	NCCN/ASCO/ESMO/CSCO
RecurIndex®	28	RT-PCR	HR positive, HER2 negative BC，Stage N0–N2	local recurrences: low risk < 27 high risk ≥ 27 distant recurrences: low risk < 33 high risk ≥ 33	Direct adjuvant chemotherapy or radiotherapy	China	FFPE	CSCO

The NSABP B⁃20 study showed that Oncotype DX**®** can also predict chemotherapy sensitivity. The high-risk group benefited from adjuvant chemotherapy to a greater extent than the low-risk group. The degree to which the intermediate-risk group benefited from adjuvant chemotherapy was indeterminate. However, the possibility that this treatment group could nonetheless benefit from adjuvant chemotherapy cannot be ruled out.^22^ The TAILORx trial compared 4,854 axillary node-negative patients with BC who were administered et al. one against 4,573 others who received ET plus chemotherapy. For patients with HR+ early BC and 21-gene recurrence scores in the range of 11–25, the efficacy of adjuvant endocrine therapy for ≥5 years was not inferior to that of combined chemotherapy + ET. Thus, most women with ER+, HER2-, and node-negative BC can safely avoid adjuvant chemotherapy.^14^ The RxPONDER trial compared the efficacy of ET with chemotherapy against that of Et al.one in patients presenting 1–3 positive axillary lymph nodes (ALN). In contrast, there was no significant benefit of combined therapy for postmenopausal patients with RS (0–25). In premenopausal patients, however, adding chemotherapy increased DFS at 5 years by 5.2%. Nevertheless, RS was not associated with chemotherapy efficacy.^11^ Oncotype DX**®** was validated in prospective clinical trials on premenopausal and postmenopausal patients with HR+/HER2- node-negative and node-positive BC. Oncotype DX**®** is now widely recommended for clinical use in patients with HR+/HER2-/pT1-2N0-1 early-stage BC.

### MammaPrint®

MammaPrint**®** is a transcriptome-like sequencing method consisting of 70 gene markers. Which are involved in all biological processes related to tumor progression and metastasis, and encompass the five well-defined hallmarks of cancer (supplementary table S2).Further stratification of MammaPrint® risk outcomes reveals the presence of two distinct risk subgroups: high-risk and low-risk, each exhibiting specific prognostic and predictive implications. It classifies patients with early-stage BC as those with 5-year and 10-year recurrence prognoses.^17,23^ In the MINDACT study, of 1,000 patients with ultra-low-risk 70-gene signatures and median follow-ups of 8.7 years, 97.0% exhibited an 8-year distant metastasis-free interval.^24^ The MINDACT study also demonstrated that when the clinical risk is low, chemotherapy will afford no benefit even when the gene risk is high. The benefit of chemotherapy is also very limited for patients with high clinical risk and low gene risk.^17^ The prognostic accuracy of MammaPrint**®** has been confirmed for node-negative patients but only sparsely investigated for node-positive patients.^25–27^ MammaPrint® has been applied in United States, Canada, Spain, Middle East etc. for decision-making in early-stage HR+/HER2- breast cancer.

### RecurIndex®

RecurIndex**®** was proposed by scientists in Taiwan, China and validated in Asian populations.^28–31^ The recurrence risk score may be high or low and is based on 18 core genes and clinical factors (supplementary table S5). One early study showed that the 18 core genes benefited patients with 1–3 positive ALNs. For the low-risk group, the 5-year local recurrence-free rates were in the range of 95.2–100%. The distant metastasis-free survival (DMFS) and the OS rates were in the ranges of 76.6–95.1% and 77.6–95.6%, respectively. For the high-risk group, the 5-year local recurrence-free, DMFS, and OS rates were in the ranges of 27.3–50.8%, 22.2–22.7%, and 26.7–44.4%, respectively.^32^ Option RecurIndex is more suitable for Chinese patients. Most of the other products tested are imitations, and their effectiveness is questionable.

RecurIndex^**®**^ is used mainly to predict the risk of distant metastasis in patients with stage N0–2 BC who have already undergone surgery. It is also applied to establish the potential benefits of chemotherapy for various patients. A validation study comprised 752 patients with operable stage I – III invasive BC and a median follow-up time of 95.8 months. The patients were divided into low-risk and high-risk groups according to their RecurIndex**®** scores, were not at risk of distant metastasis for 10 years, and had disease recurrence-free interval (DRFI) rates of 94.1% and 85.0%, respectively (*p* < .0001). Their 10-year recurrence-free (relapse-free) survival (RFS) rates were 90.0% and 80.5%, respectively (*p* = .0003).^33^ A subgroup analysis showed that the 10-year DRFI rates for the low-risk patients in the chemotherapy and no-chemotherapy groups were 97.0% and 93.4%, respectively, while those for the high-risk patients in the chemotherapy and no-chemotherapy groups were 85.2% and 82.3%, respectively.^33^ A subsequent study demonstrated that for 490 high-risk and low-risk patients with HR+/HER2- BC, the difference in DRFI was significant (*p* < .05) whether the patients were positive or negative for lymph node metastasis.^34^ A multivariate analysis confirmed that the RecurIndex**®** model can independently predict DRFI (HR = 6.76, *p* < .0001) and OS (HR = 6.06, *p* = .01) for both high-risk and low-risk patients.^34^ Hence, the RecurIndex**®** score has prognostic value and may help improve the efficiency of postoperative adjuvant chemotherapy administration.

### EndoPredict®

EndoPredict® comprises four normalization and control genes, along with eight target genes associated with proliferation and the ER pathway^35^(supplementary table S3).which combines transcriptomic and clinical risk factors, test results are given as an EPclin Risk Score, and categorize patients as those at high or low risk of recurrence.^36^ It can be implemented for postmenopausal patients with either node-positive or node-negative BC.^36,37^ EndoPredict**®** is prognostic when it is applied to assess late distant recurrence after 5–15 years in patients who have not relapsed after 5 years.^37^ EndoPredict now available in Europe. Overall, several gene expression assays are available to estimate the distant risk of recurrence in early-stage, HR+/HER2- BC and are fully interchangeable.^38^ Future trials should compare their relative effectiveness at predicting the risk of recurrence in BC risk.

## Application of multigene testing in postoperative extended ET for patients with stage T1–2N0–1 early invasive BC

### Breast cancer index (BCI)

The Breast Cancer Index (BCI) is a gene expression-based signature that focuses on estrogen and proliferative signaling. BCI Testing is available in all 50 states of America.BCI combines the HOBXB13/IL17BR score (H/I) and the molecular grade index.^39,40^ The molecular grade index is a gene expression assay that consists of five genes associated with histological grade and tumor progression (supplementary table S4). By utilizing a combinatorial approach involving H: I and MGI, it has been demonstrated that breast cancer patients can be categorized into three risk groups.^41^ It estimates the 10-year relapse risk and the benefit of long-term adjuvant ET.^27,42^ The Trans-aTTOM study included 789 patients with HR+, node-positive BC. Compared to the patients in the HER2- subgroup who were treated with tamoxifen for 5 years, those who were treated for 10 years had a significantly longer recurrence-free interval (RFI), a higher BCI (H/I), and a longer disease-free interval (DFI) (HR = 0.35, *p* = .047; HR = 0.41, *p* = .047). However, they did not significantly differ in terms of lower BCI (HR = 1.15, *p* = .491; HR = 1.10, *p* = .612).^43^ Hence, prolonged ET may benefit those with high BCI. There was also no significant difference between subgroups in terms of the ability of their HER2- status to predict BCI (*p* = .849).^43^ The latter was then validated in the IDEAL study which evaluated the benefit of extending 5-year letrozole therapy by another 5 years vs. 2.5 years.^2^ It was proposed that extended ET may be indicated for patients at high risk of BCI regardless of their clinical risk assessment. This hypothesis was validated in several subsequent prospectively designed retrospective studies. Nevertheless, certain validation studies showed that its efficacy in predicting the benefit of extended adjuvant ET benefit was not statistically significant.^44–46^

### EndoPredict®

The EndoPredict assay was developed to combine genomic and clinical data, incorporating clinico-pathological factors like tumor size and nodal status. The long-term prognostic value of EndoPredict**®** for patients with ER+, HER2- BC was verified in the ABCSG6&8 study. Analyses of the 15-year prognoses demonstrated that for all postoperative patients, the 15-year DRFS rates were 93.4% and 74.6% for the EPclin low-risk and EPclin high-risk groups, respectively (*p* < .001). For patients who did not relapse within the year, the 5 ~ 15-year DRFS rates were 95.7% and 84.1% for the EPclin low-risk and EPclin high-risk groups, respectively (*p* < .001).^37^ Studies suggest that extended ET might be indicated for patients in the EPclin high-risk group.

## Application of liquid biopsy in postoperative extended ET for patients with stage T1–2N0–1 early invasive BC

Exosomes, circulating tumor DNA (ctDNA), circulating tumor cells (CTCs), cell-free RNA (cfRNA), and different liquid-based biomarkers substances are formed by apoptosis, necrosis, and other processes and released into the blood during malignant tumor formation and growth.^47,48^ The present review focuses mainly on the advantages, challenges, and clinical utility of CTCs and ctDNA testing in patients with early-stage HR+ BC.

Minimal residual disease (MRD) is a condition in which small quantities of tumor cells remain in patients who have already undergone treatment. MRD indicates a poor prognosis. Sanger-based ctDNA detection methods were used for MRD monitoring. However, they have low throughput and a limited detection range. Therefore, more precise and convenient assays such as quantitative polymerase chain reaction (qPCR), digital PCR (dPCR), single-cell sequencing (SCS), and next-generation sequencing (NGS) were developed for this purpose.^49,50^ MRD monitoring has been applied mainly for solid tumors. Several large prospective, observational studies have demonstrated that ctDNA status is a significant prognostic biomarker for colorectal cancer (CRC) and lung cancer, which may guide treatment approaches for them. More recently, studies have been conducted on the implementation of ctDNA status in the prognostication of early-stage primary BCs.

### ctDNA detection

Patients with early-stage BC who present MRD after surgery or systemic therapies are at a relatively high risk of recurrence. Clinical studies have shown that the median lead time between ctDNA detection and relapse in HR+ BCs is in the range of 6.5–12.4 months.^18,51,52^

Garcia-Murillas et al.^52^ were one of the first researchers to demonstrate that when ctDNA is detected in the plasma of patients with BC who have undergone surgery, the median relapse rate is eight months. That study enrolled 55 patients who were diagnosed with early-stage BC and were administered neoadjuvant chemotherapy before surgery. Of these, patient A310006 had detectable post-surgery ctDNA. This elevated mutation level suggests that the ctDNA status is associated with the disease burden. Three separate subclonal nondriver mutations that are lost in the MRD repopulate metastatic recurrence. Thus, driver mutations should be preferentially tracked in this scenario. In three patients, the metastatic relapse was restricted to the brain, and no ctDNA was detected at follow-up. For these reasons, it might be unfeasible or impracticable to utilize mutation tracking to identify recurrences limited to the central nervous system (CNS).

Updated findings of the PENELOPE-B study were presented at the 2023 annual meeting of the American Association for Cancer Research (AACR). Seven patients with detectable post-surgery ctDNA were then administered 1 year of palbociclib adjuvant to ET. Of these, two patients had no detectable ctDNA by cycle seven. Hence, there is an association between ctDNA clearance and adjuvant therapy.^53^

The foregoing studies suggest that ctDNA is a prognostic and predictive biomarker for BC recurrence and therapeutic response, respectively. Certain researchers have launched prospective studies assessing the role of ctDNA status in personalized treatment strategies. The TRAK-ER (NCT04985266) trial investigates whether therapy escalation may prevent clinical recurrence in patients with HR+ BC and ctDNA-based molecular relapse. Other studies such as DARE (NCT04567420) and LEADER (NCT03285412) prospectively implement ctDNA to select patients and correlate their ctDNA status with their clinical outcome and treatment responses. Future research should explore the potential of ctDNA-guided intervention to modify the clinical outcome of patients with HR+ BC.

The most widely investigated liquid biopsy marker is ctDNA-based monitoring. Whereas the association between ctDNA status and cancer prognosis is clear, ctDNA detection is challenging for brain and bone metastases. False negatives may arise because of low sensitivity while false positives might occur in response to clonal hematopoiesis. Thus, mutations should be preferentially tracked. The aforementioned challenges notwithstanding, ctDNA-based monitoring holds promise as a crucial component in the clinical management of early-stage BC.

### Circulating tumor cells (CTCs)

CTCs are released into the bloodstream from primary tumor masses. In early-stage HR+ BC, they serve as diagnostic and prognostic indicators.^54^

Fischer et al.^55^ reported a solid relationship between CTC level and cancer prognosis. They associated the detection of CTCs after adjuvant chemotherapy with reduced overall survival (OS) and disease-free survival (DFS). Hence, CTCs could be implemented as prospective biomarkers guiding and monitoring the administration of adjuvant therapy.

A retrospective analysis^56^ showed that CTC status was associated with the clinicopathological characteristics of early-stage BC. Patients with detectable CTC at baseline (*n* = 483; 39.6%) had comparatively larger tumors (Pearson’s χ^2^ *p* = .010), more frequent positive nodal

status (Pearson’s χ^2^ *p* = .028), and higher Ki-67 expression (Pearson’s χ^2^ *p* = .001). The authors also assumed that post-therapy CTC persistence is a potential marker of resistance to adjuvant treatment.

## Application of multigene testing in postoperative adjuvant radiotherapy for patients with stage T1–2N1 early invasive BC

There is an urgent clinical requirement to screen patients with stage N1 BC to determine whether they require postoperative adjuvant radiotherapy.

### RecurIndex®

A recent study was conducted on 388 patients with stages I – III, N1–2 BC who had undergone either radical mastectomy or breast-conserving surgery. The 10-year local recurrence-free interval (LRFI) rate for the patients in the low-risk group was 100% whether or not they were administered radiotherapy. Hence, they do not necessarily benefit from this treatment modality. For the patients in the high-risk group who received radiotherapy and for those who did not, the 10-year LRFI rate was 100% whereas the annual LRFI rates were 93.7% and 75.5%, respectively.^57^ Thus, radiotherapy may reduce the risk of local and regional recurrence in the high-risk group. The researchers assessed the clinical value of RecurIndex**®** in decision-making for postmastectomy radiotherapy (PMRT) in patients with stage pT1–2N1M0 BC. The 7-year LRFI, DRFI, and RFS for the low-risk patients (96.14%, 92.28%, and 88.55%, respectively) were higher than those for the high-risk patients (84.30%, 82.03%, and 73.83%, respectively).^58^ Subgroup analysis showed that there were no significant differences in 7-year LRFI, DRFI, RFS, and OS between low-risk patients who received PMRT and those who did not. However, the 7-year LRFI for high-risk patients who received PMRT and those who did not were 87.87% and 74.87%, respectively (*p* = .0071). Under the same grouping conditions, the 7-year DRFI, RFS and OS were 86.96% and 69.16% (*p* = .019), 86.96% and 69.16% (*p* = .019), 79.41% and 59.46% (*p* = .019), 88.26% and 70.27%(*p* = .014), respectively.^58^ The RIGAIN study (NCT04069884) is a multicenter, open-label, randomized, controlled, prospective phase III trial designed to explore whether whole breast irradiation plus regional lymph node irradiation (RNI) after breast-conserving surgery or chest wall irradiation and RNI after mastectomy can improve clinical outcomes in N1 patients with a low clinical risk of LRR but a high risk of LRR as determined by RecurIndex.^59^ In addition, the IMNI PRECISION trial is a phase II, open-label, noninferiority randomized controlled trial (NCT04517266). The design allows a promising clinicogenomic model (RecurIndex) to stratify individual risk of developing relapse and guide optimal combined regional nodal irradiation (RNI) and internal mammary lymph node irradiation (IMNI) in patients with early-stage (pT1-2N1) BC.^59^ We look forward to the results of these two prospective studies.

When the RecurIndex**®** test shows that patients with N1 stage BC are at low risk, postoperative local and regional radiotherapy may be contraindicated. In contrast, when the RecurIndex**®** test shows that patients with N1 stage BC are at high risk, postoperative adjuvant radiotherapy may be indicated to lower the risk of local and regional recurrence.

### Oncotype DX®

The NSABP B-28 study disclosed that the 10-year cumulative incidence rates of locoregional recurrence in low-risk, intermediate-risk, and high-risk patients with BC were 3.3%, 7.2%, and 12.2%, respectively (*p* < .001). It also revealed that RS is an independent predictor of locoregional recurrence (HR = 2.59, *p* = .008).^60^ Therefore, as Oncotype DX**®** indicates the risk of locoregional recurrence, it can screen patients with HR+, node-positive BC requiring postoperative adjuvant radiotherapy. A retrospective study was conducted on RS in 316 postmenopausal patients with HR+, node-positive early BC. The 10-year locoregional recurrence rates in the low-risk group and the combined intermediate- and high-risk groups were 9.7% and 16.5%, respectively (*p* = .02). A multivariate analysis showed that the risk of locoregional recurrence was 2.36 times higher in the intermediate- and high-risk patients than in the low-risk patients (*p* = .04).^61^ An analysis of patients who had undergone mastectomy but not radiotherapy and presented 1–3 lymph node metastases showed that the locoregional recurrence rates for the low-risk and the combined intermediate- and high-risk patients were 1.5% and 11.1%, respectively (*p* = .051).^62^

Hence, Oncotype DX**®** may be somewhat useful for assessing the risk of locoregional BC recurrence. It also indicates that patients in the low-risk group may be exempted from postoperative adjuvant radiotherapy.

## Application of CTC in postoperative adjuvant radiotherapy for patients with stage T1–2N1 early invasive BC

Goodman et al.^62^ assessed the identification of CTC status and prediction of the benefits of radiotherapy in patients with early-stage BC. For those in the CTC-positive cohort who had undergone breast-conserving surgery, radiotherapy was associated with longer OS. However, there was no difference in OS between the CTC-negative and CTC-positive patients who underwent radical mastectomy. The foregoing findings suggest that evaluating CTC status following BC resection could be informative.

### Adjuvant immunotherapy in high-risk, HR+, early-stage BC

BC used to be regarded as immunogenically ‘cold’. Nevertheless, two immune checkpoint inhibitors (ICIs) are currently approved for use in combination with chemotherapy for PD-L1-positive advanced triple-negative breast cancer (TNBC). The efficacy of ICIs in the management of TNBC led to the implementation of similar therapeutic approaches for hormone receptor-positive (HR+)/HER2- BC. Early research on HR+/HER2- BC explored the administration of ICI monotherapy against metastatic disease. However, more recent studies showed that this strategy elicited only temporarily durable responses and had limited efficacy. Though early-stage HR+/HER2- BCs have a better prognosis than triple-negative BCs, the pathological complete response (pCR)-to-neoadjuvant chemotherapy (NACT) conversion rates are still low. Several clinical trials evaluated the efficacies of various neoadjuvant immunotherapy combinations and their impact on the pathologic response. Nonetheless, long-term outcome data are lacking.

Checkmate 7FL is the first prospective, phase 3, randomized trial comparing the efficacies of nivolumab (NIVO) + NACT + adjuvant ET (Arm A) and placebo + NACT/ET (Arm B) against high-risk, early-stage ER+ HER2- BC. Arm A had a higher overall pCR (24.5%) than Arm B (13.8%). At stage III, the pCR for Arms A and B were 30.7% and 8.1%, respectively (*p* = .0021). NIVO treatment significantly improved pCR in PD-L1-positive patients. The overall AD was 24.1%, and the ADs were 44.3% and 20.2% for the patients in Arms A and B, respectively.^63^ The efficacy of NIVO increased with PD-L1 expression. Patients with combined positive scores (CPS) > 1 had 16.6% ∆pCR (unweighted rate difference between groups A and B) (group A/B: 40.4% vs. 23.8%; 95% CI: 2.8–29.4). Patients with CPS ≥ 10 had 32.4% ∆pCR (group A/B: 65.7% vs. 33.3%; 95% CI: 7.3–52.3). Patients with CPS ≥ 20 had 52.3% ∆pCR (group A/B: 78.9% vs. 26.7%; 95% CI: 18.6–72.4).

In contrast to Checkmate7FL, KEYNOTE-756 has two primary endpoints, namely, pCR and event-free survival (EFS). In a global phase 3 trial, patients with early-stage, high-risk ER+/HER2- malignant BC were administered either neoadjuvant pembrolizumab or placebo + chemotherapy followed by ET. Centrally confirmed grade 3 invasive ductal ER+/HER2- malignant BC patients (T1c-2 (≥2 cm), cN1-2, or T3–4 cN0-2) were randomized 1:1 to receive either neoadjuvant pembrolizumab 200 mg Q3W or placebo. Patients were administered paclitaxel QW for 12 weeks followed by four cycles of doxorubicin or epirubicin + cyclophosphamide (neoadjuvant treatment). The final pCR analysis at the first interim data cutoff was conducted on May 25, 2023, and disclosed a median follow-up period of 33.2 months (range: 9.7–51.8 months). For the intention-to-treat (ITT) population, pembrolizumab + chemotherapy improved pCR (ypT0/Tis ypN0) to a significantly higher degree than placebo + chemotherapy. The secondary pCR definitions and their respective values were ypT0 ypN0 (21.3% vs. 12.8%) and ypT0/Tis (29.4% vs. 18.2%).^63^ The prevalence of ≥ grade 3 drug-related side effects in response to the sandwich regimen was ~ 45%. Therefore, the neoadjuvant + adjuvant approach may be excessive. Other assessment tools should be implemented going forward to identify the patients that are most suitable for the sandwich regimen.

Nivolumab and pembrolizumab are perioperatively administered in cases of HR+ early BC at a significantly high risk of recurrence. However, the overall pCR rate in the ITT population is a suboptimal ~ 25%. PD-L1 expression is a reliable efficacy index according to the subgroup analysis. Other biomarkers of immunotherapy efficacy against HR+ BC are urgently needed. The molecular characterization of breast tumors has improved their clinical treatment. METABRIC integrates the subtyping of BC-based copy number alterations (CNAs) and gene expression in primary tumors and has revealed ten integrative clusters (IntClust 1–10).^64^ The Cancer Genome Atlas (TCGA) identified four major breast cancer subtypes via multiplatform clustering.^65^ Shao et al.^66^ assembled a large-scale multi-omics cohort comprising 579 patients with HR+/HER2- BC and classified them as canonical luminal, immunogenic, proliferative, and receptor tyrosine kinase (RTK)-driven molecular subtypes. As the immunogenic subtype presented an abundance of immune cells, it could be more responsive to ICI than the other three subtypes.

Tumor-infiltrating lymphocytes (TILs) may be vital to antitumor immunity.^67^ A high abundance of stromal TILs (sTILs) indicates survival benefit in primary TNBC and HER2+ BC. However, their significance in HR+/HER2- BC is unknown.^68^ Luminal BC is the least immunogenic subtype of all BCs.^69^ High TIL abundance is associated with relatively better distance disease-free survival (DDFS)^69^ and prognosis.^70^ However, the role of TILs in HR+/HER2- BC remains unknown and might be elucidated through future prospective cohort studies.

## Discussion

Breast tumors exhibit significant heterogeneity in both morphology and molecular profiles, influenced by various factors that affect treatment response and recurrence risk.^71,72^ Multigene molecular tests leverage advanced technologies, such as quantitative PCR – utilized in assays like Oncotype DX, EndoPredict, Breast Cancer Index (BCI), and Recurrence Index – as well as microarray techniques exemplified by MammaPrint.^73–75^ These tests analyze a wide range of genes, as detailed in supplementary Tables S1-S5. Although the gene lists vary among these assays, some genes overlap, such as BIRC5, which is present in both Oncotype DX and EndoPredict, and ESR1, found in both Oncotype DX and the Recurrence Index. Importantly, each testing tool employs distinct algorithms and thresholds for estimating recurrence probabilities, categorizing patients into low, intermediate, or high-risk groups. To enhance the accuracy of risk predictions, it is crucial to integrate these multigene risk scores with additional clinical parameters, including tumor size, nodal status, tumor grade, and patient age or menopausal status. Only some small sample observation study, for instance, A comparative study involving female Taiwanese patients with breast cancer demonstrated that the recurrence index for distant recurrence (RI-DR) and Oncotype DX Recurrence Score (ODx RS) exhibited high consistency in performance. Both RI-DR and ODx RS were similarly effective in predicting recurrence-free survival (RFS) in patients with early-stage breast cancer.^76^ However, the existing literature lacks large-scale, head-to-head comparisons of multigene assays, making it challenging to determine the most effective approach for tailoring adjuvant systemic therapy in early-stage hormone receptor-positive (HR+) and HER2-negative (HER2-) breast cancer. Each assay presents its own body of evidence supporting its clinical utility; however, without direct comparisons, drawing definitive conclusions about their relative effectiveness remains difficult.

The NCCN guidelines recommend considering transcriptomic testing for all patients with invasive ductal or lobular tumors greater than 0.5 cm in diameter and no lymph node involvement, and for patients with 1–3-node-positive disease who are candidates for adjuvant chemotherapy.^77^ For node-negative patients, premenopausal women may still require chemotherapy despite low to intermediate Oncotype DX Recurrence Scores (RS), as their risk factors can be multifaceted. In contrast, postmenopausal women with an RS below 25 are typically classified as low-risk and can often safely forgo chemotherapy, as demonstrated by the TAILORx trial.^78^ In node-positive cases, particularly those with 1–3 positive lymph nodes, Oncotype DX offers critical prognostic information, indicating that high-risk postmenopausal patients can achieve significant survival benefits from chemotherapy. MammaPrint is also applicable for patients with pN0 and pN1 clinical high-risk disease, as it stratifies patients into risk categories, allowing clinicians to identify those with Ultra-Low Risk tumors who can avoid chemotherapy while maintaining excellent survival rates.^79^

As there is a high probability of late recurrence in HR+ BC, several studies have aimed to determine the most efficacious ET duration. Adjuvant ET is the primary approach for the control of early luminal-like BC. EndoPredict combines molecular factors with tumor size and nodal status to comprehensively assess recurrence risk, identifying patients who may benefit from EET. BCI provides prognostic information on overall and late distant recurrence risk, as well as predictive data on the likelihood of EET benefit. Patients classified as low-risk by EPclin or BCI Prognostic may safely forgo extended therapy, while high-risk patients predicted to benefit from EET by BCI H/I could consider EET, weighing the potential absolute risk reduction against the risks of prolonged treatment.^2,80^

Research in the area of oncological liquid biopsy is rapidly expanding. It states that the prognosis may be poor for patients with early-stage BC and CTC < 1/7.5 mL serum. CTCs are prognostic tools for BC, and the biological indices ER, PR, HER2, and Ki-67 are applied in the histological grading of tumors. In 2019, the Chinese Society of Clinical Oncology (CSCO) included CTCs in their therapeutic and diagnostic guidelines for BC. The prognostic relevance of CTCs in many types of solid cancer has been empirically demonstrated. The changes in CTC counts that occur during therapy can predict the treatment response. However, CTC analyses require standardization and quality control as CTCs are highly heterogeneous. Moreover, few hospitals have introduced or conducted genetic testing projects, the costs of the latter are high, and patients must pay for them out of pocket. For these reasons, certain patients question the merit or value of such procedures. It is hoped that the cost of genetic testing may be reduced and that insurance will cover it in the future.

Several emerging technologies have the potential to significantly enhance risk stratification soon. Systemic recurrence following surgical resection of the primary tumor, despite the absence of detectable disease, indicates that a small number of disseminated cancer cells survived adjuvant treatment and may lead to recurrence years later. Though liquid biopsies are far more promising than conventional test programs, they are nonetheless associated with significant challenges. The escalation of personalized adjuvant and consolidation therapy may lower the risk of toxicity and raise the cure rates by incorporating ctDNA-MRD testing into conventional clinical practice. Liquid biopsy biomarkers can effectively forecast and discriminate the risks and benefits related to various therapeutic modalities. Nevertheless, it remains to be established whether these blood markers are interchangeable or complementary. It must also be determined which biomarkers present first and which ones play the most important roles during BC recurrence. As molecular biomarker and metastasis detection methods have not yet been standardized, technological advancements are required to improve the performance of liquid biopsy. Going forward, extensive prospective studies should be conducted. Risk-monitoring tools such as liquid biopsy ctDNA-MRD should be implemented to assess the risk of recurrence in patients with BC and enable clinicians to make effective and precise treatment decisions. Despite advancements in risk stratification, several questions remain unanswered in early-stage breast cancer.

In summary, multigene assays primarily focus on early-stage, HR-positive, HER2-negative breast cancer patients. They provide essential information for treatment decisions, including the prediction of chemotherapy benefits, assessment of recurrence risk, and guidance for adjuvant therapy. However, significant questions remain regarding variations in racial demographics and access to multigene assays across different regions. While these tools demonstrate strong predictive accuracy for disease recurrence and overall survival, the optimal choice among them remains uncertain. To effectively assess the risk of recurrence in HR-positive, HER2-negative breast cancer, it is crucial to routinely evaluate disease stage, biology, and genomic characteristics. This comprehensive assessment, potentially augmented by artificial intelligence algorithms, could enhance our ability to identify patients who would benefit from systemic therapy. Additionally, emerging technologies such as liquid biopsies and innovative techniques like mRNA and methylation analysis should be further explored to improve clinical decision-making.

## Authors’ contributions

Conception and design: Ying Xu, Yingxue Qi.

Acquisition of data (download publications, tables): Zhongyu Lu, Yuan Tan.

Writing, review, and/or revision of the manuscript: Ying Xu, Yingxue Qi, Zhongyu Lu, Yuan Tan, Dongsheng Chen, Haijun Luo.

## Supplementary Material

Supplementary table 3 Gene list of EndoPredic.xlsx

Supplementary table 1 Gene list of Oncotype DX.xlsx

Supplementary table 5 Gene list of RecurIndex.xlsx

Supplementary table 2 Gene list of MammaPrint.xlsx

Supplementary table 4 Gene list of BCI.xlsx
